# A mechanism for bistability in glycosylation

**DOI:** 10.1371/journal.pcbi.1006348

**Published:** 2018-08-03

**Authors:** Andrew G. McDonald, Keith F. Tipton, Gavin P. Davey

**Affiliations:** School of Biochemistry and Immunology, Trinity College Dublin, Dublin, Ireland; University of California San Diego, UNITED STATES

## Abstract

Glycosyltransferases are a class of enzymes that catalyse the posttranslational modification of proteins to produce a large number of glycoconjugate acceptors from a limited number of nucleotide-sugar donors. The products of one glycosyltransferase can be the substrates of several other enzymes, causing a combinatorial explosion in the number of possible glycan products. The kinetic behaviour of systems where multiple acceptor substrates compete for a single enzyme is presented, and the case in which high concentrations of an acceptor substrate are inhibitory as a result of abortive complex formation, is shown to result in non-Michaelian kinetics that can lead to bistability in an open system. A kinetic mechanism is proposed that is consistent with the available experimental evidence and provides a possible explanation for conflicting observations on the *β*-1,4-galactosyltransferases. Abrupt switching between steady states in networks of glycosyltransferase-catalysed reactions may account for the observed changes in glycosyl-epitopes in cancer cells.

## Author summary

While enzymes tend to have a narrow substrate specificity, there are a number of enzymes that are promiscuous, acting on a wide range of substrates. In this article we derive expressions for general multi-substrate competitive inhibition for the class of transferases, with particular emphasis on glycosylation. By extending the enzyme reaction mechanism to include inhibition by high substrate concentrations, we show that switching behaviour (bistability) is possible within a thermodynamically open systems of glycosylation enzymes. The biological implication of this finding is that small changes to a predictor variable may induce abrupt changes in the secreted products.

## Introduction

With the ready availability both of computing power and software tools for numerical simulation, the mathematical modelling of metabolic systems has become a core component of cell biology. Models of classical metabolic pathways, such as glycolysis [1–3], the citric-acid cycle [4], the urea cycle [5] and biosynthetic pathways such as N-linked and O-linked glycosylation [6, 7], have been developed as a way to understand how such processes are regulated. Online repositories of such models, such as the BioModels database [8], allow many of these models to be examined without the need for programming ability on the part of the user. Software such as E-Cell [9] have enabled more complex models to be constructed at the cellular or organelle level.

This paper examines a particular class of metabolic model, in which one or more enzymes can act on multiple substrates. To this class belong the cytochrome P450 enzymes that are involved in detoxifying multiple xenobiotics [10], ribonuclease P [11] and also the enzymes of N-linked glycosylation [12–15]. Such enzymes recognise multiple substrates, and the products of the reactions can themselves become substrates, thus introducing a form of competitive inhibition with catalysis. It is known that, in the case of two substrates acted upon by the same enzyme the Michaelis constant of the kinetic rate law will be modified to include the effects of competing substrates upon one another [16, 17]. In the first part of this paper, a general form of the Michaelis-Menten equation for *n* competing substrates is derived, and extended to an ordered-sequential mechanism involving a donor molecule held in common by all reactions. In the second part, we model galactosyltransferase acting on an initial acceptor glycoprotein to form two products, each of which are substrates for the same enzyme. Here we propose a possible mechanism for such behaviour and apply it to the glycosyltransferase model, demonstrating the switching between stable steady states over a range of parameter values.

## Methods

### Theoretical development

**Fig 1:**
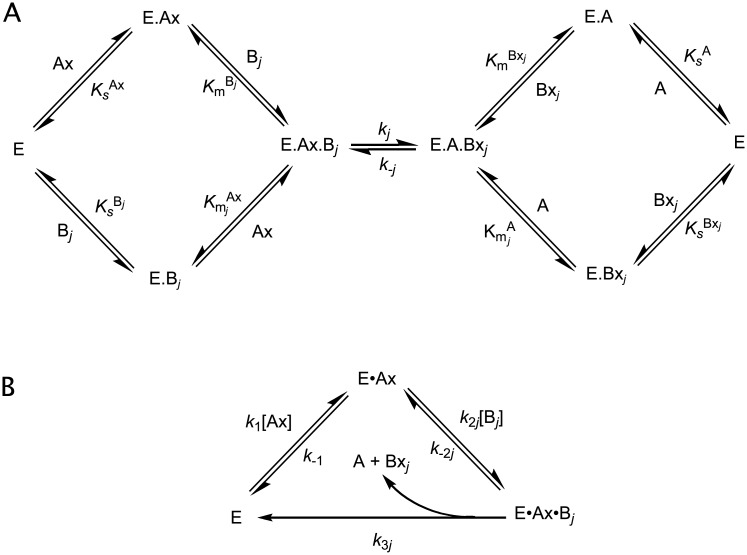
Enzyme mechanisms. A. Random-order addition of substrates, under reversible, rapid-equilibrium conditions. B. Compulsory-order addition of substrates, with quasi-steady-state assumptions.

Consider the case of a general two-substrate enzyme mechanism, in which a donor molecule, Ax, transfers the x moiety to an acceptor, B,

$$\begin{array}{c} \text{Ax}+\text{B}\to \text{A}+\text{Bx}, \end{array}$$

a reaction type that is common to the transferases. We consider the situation in which there are *n* acceptor substrates, B_1_ … B_*n*_. For random-order binding of donor and acceptor (Fig 1A), an expression for the initial rate of appearance of the *j*th acceptor product, Bx_*j*_, is

$$\begin{array}{c} \begin{array}{cc} v_{j} & =\frac{V_{j}\left[\text{Ax}\right]\left[{\text{B}}_{j}\right]}{\begin{array}{c} K_{s}^{\text{Ax}}K_{\text{m}}^{{\text{B}}_{j}}\left(1+s_{{\text{B}}_{j}}^{\prime }\right)+K_{\text{m}}^{{\text{B}}_{j}}\left[\text{Ax}\right]\left(1+s_{{\text{B}}_{j}}\right)+K_{{\text{m}}_{j}}^{\text{Ax}}\left[{\text{B}}_{j}\right]+\left[\text{Ax}\right]\left[{\text{B}}_{j}\right] \end{array}} \end{array} \end{array}$$ (1)

where *V*_*j*_ = *k*_*j*_[E_0_] is the maximal velocity obtained at saturating levels of Ax and B_*j*_, $K_{{\text{m}}_{j}}^{\text{Ax}}=K_{s}^{\text{Ax}}K_{\text{m}}^{{\text{B}}_{j}}/K_{s}^{{\text{B}}_{j}}$, $s_{{\text{B}}_{j}}=\sum_{i\neq j}\left[{\text{B}}_{i}\right]/K_{\text{m}}^{{\text{B}}_{i}}$ and $s_{{\text{B}}_{j}}^{\prime }=\sum_{i\neq j}\left[{\text{B}}_{i}\right]/K_{s}^{{\text{B}}_{i}}$. The derivation of this equation under rapid-equilibrium conditions is given in the S1 Appendix. In this model the $K_{s}^{\text{Ax}}$ and $K_{s}^{{\text{B}}_{i}}$ are, respectively, the individual dissociation constants of Ax and B_*i*_ from the E⋅Ax and E⋅B_*i*_ enzyme-substrate complexes, while the Michaelis constants of these species, $K_{{\text{m}}_{j}}^{\text{Ax}}$ and $K_{\text{m}}^{{\text{B}}_{i}}$ are the corresponding dissociation constants of the E⋅Ax⋅B_*i*_ complex. The $s_{{\text{B}}_{j}}$ and $s_{{\text{B}}_{j}}^{\prime }$ terms are sums of dimensionless acceptor substrate concentrations representing the degree to which the enzyme is competitively inhibited by substrates other than B_*j*_ itself. In the absence of substrate competition, $s_{{\text{B}}_{j}}=0$, and Eq (1) reduces to the standard form of a bisubstrate enzyme mechanism. In the limit, as [Ax] → ∞, (1) becomes

$$\begin{array}{c} v_{j}=\frac{V_{j}\left[{\text{B}}_{j}\right]}{K_{\text{m}}^{{\text{B}}_{j}}(1+s_{{\text{B}}_{j}})+\left[{\text{B}}_{j}\right]}, \end{array}$$ (2)

an equation that is similar in form to that obtained in other studies [14, 18, 19].

Although the $s_{{\text{B}}_{j}}$ symbolism is a convenience in order to show which terms of the rate law are affected by competitor concentrations, a representation that is more useful in computer simulations is the sum of concentrations of all its substrates, each weighted by its $K_{\text{m}}^{{\text{B}}_{j}}$ or $K_{s}^{{\text{B}}_{j}}$:

$$\begin{array}{c} A_{\text{E}}=\sum_{i=1}^{n}\frac{\left[{\text{B}}_{i}\right]}{K_{\text{m}}^{{\text{B}}_{i}}},A_{\text{E}}^{\prime }=\sum_{i=1}^{n}\frac{\left[{\text{B}}_{i}\right]}{K_{s}^{{\text{B}}_{i}}}. \end{array}$$ (3)

Substituting into Eq (1),

$$\begin{array}{c} v_{j}=\frac{V_{j}\left[\text{Ax}\right]\left[{\text{B}}_{j}\right]}{K_{s}^{\text{Ax}}K_{\text{m}}^{{\text{B}}_{j}}(1+A_{\text{E}}^{\prime })+K_{\text{m}}^{{\text{B}}_{j}}\left[\text{Ax}\right](1+A_{\text{E}})}. \end{array}$$ (4)

Whereas a rapid-equilibrium random-order mechanism is a feature of polypeptide *N*-acetylgalactosaminyltransferase [20], sulfotransferases [21], fucosyltransferases [22] and sialyltransferases [23], with other glycosyltransferases, such as those of the *N*-acetylglucosaminyltransferase and galactosyltransferase families, the enzyme must bind the donor first, before catalysis can occur [24]. Under quasi-steady-state conditions (Fig 1B), the rate law for the compulsory order binding is (Eq S3 in S1 Appendix):

$$\begin{array}{c} v_{j}=\frac{V_{j}\left[\text{Ax}\right]\left[{\text{B}}_{j}\right]}{K_{s}^{\text{Ax}}K_{\text{m}}^{{\text{B}}_{j}}\left(1+s_{{\text{B}}_{j}}\right)+K_{\text{m}}^{{\text{B}}_{j}}\left[\text{Ax}\right]+K_{{\text{m}}_{j}}^{\text{Ax}}\left[{\text{B}}_{j}\right]+\left[\text{Ax}\right]\left[{\text{B}}_{j}\right]} \end{array}$$ (5)

In such a case, the inhibitory effect of multi-substrate competition will lessen as the concentration of the donor is increased towards saturating levels.

## Results

### Inhibition at high substrate concentrations

Thus far, the possibility of abortive (dead-end) ternary enzyme complexes has not been considered, which in random-order mechanisms are likely to occur [25]. Experimental evidence for the existence such complexes can be the appearance of inhibition at high substrate concentrations; in the case of glycosyltransferases, the inhibition is usually that of the acceptor [26–29], but can also be that of the donor [30]. If we consider only the acceptor, an examination of the mechanism (Fig 1A) reveals that four additional binding events can occur, with the E⋅Ax, E⋅B_*j*_, E⋅A and E⋅Bx_*j*_ complexes. We consider binding of B_*j*_ to the second of these complexes, E⋅B_*j*_, to provide a possible explanation for substrate inhibition with increasing acceptor concentration. Not only will B_*j*_ bind, but so will any competitive acceptor-substrate B_*i*_, *i* = 1, …, *n*.

The oligosaccharides attached to glycoproteins (glycans) can be multivalent, meaning that the same acceptor has more than one recognition domain. By way of illustration, the enzyme *β*-*N*-acetylglucosaminylglycopeptide *β*-1,4-galactosyltransferase (GalT; EC 2.4.1.38), catalyses the transfer of d-galactose (Gal) residue to a terminal *N*-acetylglucosaminyl (GlcNAc) residue on a glycoprotein, glycopeptide or polysaccharide, with the general reaction:

$$\begin{array}{c} \text{UDP-}\alpha \text{-}\text{D}\text{-Gal}+\beta \text{-}\text{D}\text{-GlcNAc-R}\to \text{UDP}+\text{Gal-}\beta 1,4\text{-}\text{D}\text{-}\beta \text{-}\text{D}\text{-GlcNAc-R} \end{array}$$

**Fig 2:**
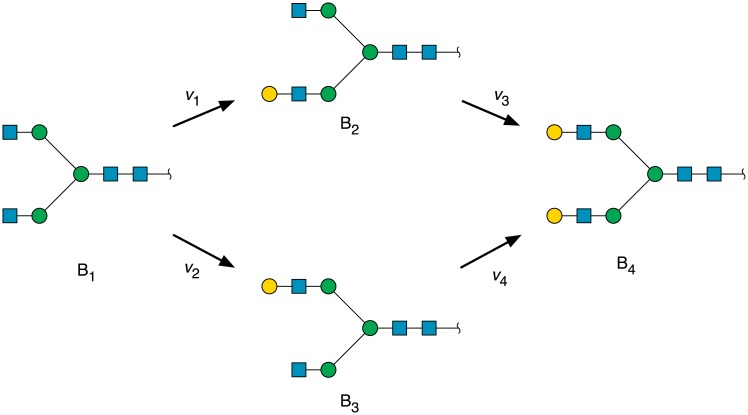
Model scheme of GalT acting on a diantennary *N*-glycan, in which species B_1_, B_2_ and B_3_ are competing substrates. Sugar symbols used, in SNFG notation [32]: blue square, GlcNAc; green circle, Man; yellow circle, Gal.

A theoretical system, similar to that studied experimentally by Paquêt and co-workers [31], is shown in Fig 2, in which galactose is incorporated into glycopeptide in four steps, starting with the initial acceptor B_1_, to form the final product with two terminal galactoses (B_4_). Hence, the products B_2_ and B_3_ are also substrates of the enzyme, since both contain a terminal GlcNAc on which it can act. All three substrates are therefore competitive inhibitors in the earlier sense, and can form a ternary complex with E⋅B_*j*_, the free terminal *β*-d-galactose in the acceptor competing with the donor, UDP-Gal [33].

### General observations on glycosyltransferase networks

Before continuing, we make the parenthetic observation that reaction networks such as those in Fig 2 follow a binomial distribution pattern in the number of acceptors at each step. If the initial substrate has *m* sites on which an enzyme can act, then the *m* immediate acceptor-products of that substrate will each have *m* − 1 available sites. There will be a reaction hierarchy based on the combinatorial filling of available sites until the final product is reached at *m* = 0, with the number of substrates at the *k*th step following the familiar *^m^C_k_* pattern,

$$\begin{array}{c} {}^{m}C_{k}=\frac{m!}{k!(m-k)!}. \end{array}$$

After *k* steps, a glycan substrate originally with *m* sites will have *m* − *k* sites remaining. The resulting network of all possible reactions, for a single acceptor possessing *m* sites at which an enzyme can act, will have *N*(*m*) nodes and E(*m*) edges, given by $N\left(m\right)=\sum_{k=0}^{m}{}^{m}C_{k}$ and $E\left(m\right)=\sum_{k=0}^{m}{}^{m}C_{k}\left(m-k\right)$. Every node, whether substrate or product, will have degree *m*, with the in-degree of a node at the *k*th step being *k* and its out-degree being *m* − *k*. The number of possible pathways from initial substrate to final product will be

$$\begin{array}{c} P\left(m\right)=\sum_{k=0}^{m}{}^{m}C_{k}k\left(m-k\right). \end{array}$$

Every glycan will have up to *m* of each type of dissociation constant, for the enzyme of which it is a substrate, product or inhibitor.

### Inhibition at high concentrations of acceptor

Extending the derivation of the rapid-equilibrium random equation in the S1 Appendix, an additional term will be required in the denominator to represent the abortive complex(es). Since there are *n* substrates, there will be *n*^2^ ways in which to form E⋅B_*k*_⋅B_*i*_. A double summation over the indices *i* and *k* will be required, giving the additional term

$$\begin{array}{c} \sum_{i=1}^{n}\sum_{k=1}^{n}\left[\text{E}\;\cdot \;{\text{B}}_{k}\;\cdot \;{\text{B}}_{i}\right]=\left[\text{E}\;\cdot \;\text{Ax}\right]\frac{K_{s}^{\text{Ax}}}{\left[\text{Ax}\right]}\sum_{i=1}^{n}\sum_{k=1}^{n}\frac{\left[{\text{B}}_{k}\right]}{K_{\text{I}}^{{\text{B}}_{k}}}\frac{\left[{\text{B}}_{i}\right]}{K_{s}^{{\text{B}}_{i}}} \end{array}$$

where $K_{\text{I}}^{{\text{B}}_{k}}$ is the dissociation constant of the *k*th acceptor from complex E⋅B_*k*_⋅B_*i*_.

The rate of appearance of the *j*th product will then be

$$\begin{array}{c} v_{j}=\frac{V_{j}\left[\text{Ax}\right]\left[{\text{B}}_{j}\right]}{\begin{array}{c} K_{s}^{\text{Ax}}K_{\text{m}}^{{\text{B}}_{j}}\left(1+s_{{\text{B}}_{j}}^{\prime }+s_{\text{I}}\right)+K_{\text{m}}^{{\text{B}}_{j}}\left[\text{Ax}\right]\left(1+s_{{\text{B}}_{j}}\right)+K_{{\text{m}}_{j}}^{\text{Ax}}\left[{\text{B}}_{j}\right]+\left[\text{Ax}\right]\left[{\text{B}}_{j}\right] \end{array}} \end{array}$$ (6)

with

$$\begin{array}{c} s_{\text{I}}=\sum_{i=1}^{n}\sum_{k=1}^{n}\frac{\left[{\text{B}}_{k}\right]}{K_{\text{I}}^{{\text{B}}_{k}}}\frac{\left[{\text{B}}_{i}\right]}{K_{s}^{{\text{B}}_{i}}}=\sum_{k=1}^{n}\frac{\left[{\text{B}}_{k}\right]}{K_{\text{I}}^{{\text{B}}_{k}}}\sum_{i=1}^{n}\frac{\left[{\text{B}}_{i}\right]}{K_{s}^{{\text{B}}_{i}}}. \end{array}$$

When *n* = 1, this reduces to

$$\begin{array}{cc} v= & \frac{V_{\text{max}}\left[\text{Ax}\right]\left[\text{B}\right]}{K_{s}^{\text{Ax}}K_{\text{m}}^{\text{B}}+K_{\text{m}}^{\text{B}}\left[\text{Ax}\right]+K_{\text{m}}^{\text{Ax}}\left[\text{B}\right]+\frac{K_{\text{m}}^{\text{Ax}}}{K_{\text{I}}^{\text{B}}}{\left[\text{B}\right]}^{2}+\left[\text{Ax}\right]\left[\text{B}\right]} \end{array}$$ (7)

The equation for the compulsory order mechanism will be identical, and the more computationally efficient representation, equivalent to Eq (4), is

$$\begin{array}{c} v_{j}=\frac{V_{j}\left[\text{Ax}\right]\left[{\text{B}}_{j}\right]}{\begin{array}{c} K_{s}^{\text{Ax}}K_{\text{m}}^{{\text{B}}_{j}}(1+A_{\text{E}}^{\prime }+s_{\text{I}})+K_{\text{m}}^{{\text{B}}_{j}}\left[\text{Ax}\right]\left(1+A_{\text{E}}\right) \end{array}} \end{array}$$ (8)

with two summation terms, $A_{\text{E}}=\sum_{i=1}^{n}\left[{\text{B}}_{i}\right]/K_{\text{m}}^{{\text{B}}_{i}}$ and $A_{\text{E}}^{\prime }=\sum_{i=1}^{n}\left[{\text{B}}_{i}\right]/K_{s}^{{\text{B}}_{i}}$.

**Fig 3:**
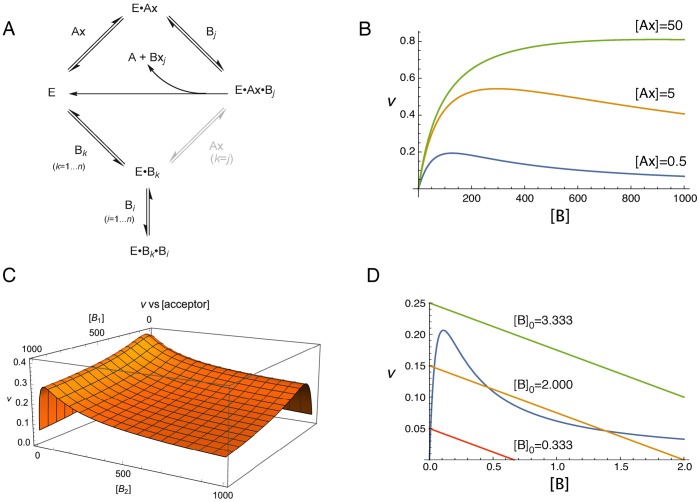
Substrate inhibition by one or more substrates. A. Reaction scheme for the formation of ternary enzyme-acceptor complex; the binding of Ax to the E⋅B_*j*_ complex (shown in grey) only occurs within the random order model. B. Substrate inhibition of an enzyme with a single acceptor, for three different donor concentrations (0.5, 5, 50) with $K_{\text{m}}^{\text{Ax}}=0.5$. C. Total enzyme initial rate as a function of two substrates, B_1_ and B_2_, exhibiting substrate inhibition through formation of an abortive (dead-end) ternary complex. D. Bistability exhibited by a substrate-inhibited enzyme (Eq (7)) in a system open to substrate, for three different values of the concentration of acceptor available externally: [B]_0_ = 0.666 (red), [B]_0_ = 2.000 (orange), [B]_0_ = 3.333 (green). At [B]_0_ = 2.0, the line intercepts the velocity–substrate curve at three points, the two outer points being stable, and the inner an unstable steady-state solution.

A general scheme for the formation of ternary enzyme-acceptor complexes is given in Fig 3A. This scheme does dual service, in illustrating both the formation of *n*^2^ inhibitory complexes in an *n*-substrate environment, but also the two catalytic mechanisms involving compulsory-order and random-order binding of substrates, which in the latter case only occurs when *j* = *k*, and for substrate inhibition at high concentrations, when *j* = *k* = *i*. The scheme illustrates two aspects of multi-substrate competition: productive, in which catalysis occurs, and non-productive, where there is inhibition as a result of abortive complex formation at higher acceptor concentrations. In the productive case, the *n* acceptors compete with each other for the E⋅Ax complex, in either random-order or compulsory-order binding mechanisms. In the non-productive case, higher acceptor concentrations compete with the donor for binding to the free enzyme, as well as with each other, for an enzyme-acceptor complex, resulting in non-productive multi-substrate inhibition in compulsory-order mechanisms. In the case of a random-order mechanism, the acceptor may bind to either the free enzyme or to the E⋅Ax complex in pathways leading to the productive ternary (E⋅Ax⋅B_*j*_) complex. Therefore high substrate inhibition may result from the mis-oriented binding of acceptor to the free enzyme or binding of a second B to the E⋅B resulting in an abortive ternary complex. The binding site at which competition occurs may differ, depending on the enzyme mechanism involved. Fig 3B displays three curves of *v* vs [acceptor], showing the relief of substrate inhibition that occurs as the donor concentration is increased, and in Fig 3C, the velocity-substrate surface defined by two $K_{\text{I}}^{\text{B}}$ values, for *n* = 2.

The situation is more complicated when multiple binding sites exist on each molecule of acceptor. According to Fig 2, B_1_ is a substrate, but B_4_ is not, while B_2_ and B_3_ can bind as substrate inhibitors, though B_1_ cannot because it does not have a terminal GlcNAc. B_4_ acts as a competitive (product) inhibitor of UDP-Gal, with two possible inhibition constants, $K_{\text{I},1}^{{\text{B}}_{4}}$ and $K_{\text{I},2}^{{\text{B}}_{4}}$. The effective value of *n* is the number of edges, *E*(*m*), in the network of a substrate with *m* recognition sites, as defined in the previous section, which gives 16 summands in *s*_I_. For the network in Fig 2, therefore,

$$\begin{array}{lllllll} s_{\text{I}} & =\frac{\left[{\text{B}}_{4}\right]}{K_{\text{I},\text{1}}^{{\text{B}}_{4}}}\frac{\left[{\text{B}}_{1}\right]}{K_{s,1}^{{\text{B}}_{1}}} & +\frac{\left[{\text{B}}_{4}\right]}{K_{\text{I},\text{2}}^{{\text{B}}_{4}}}\frac{\left[{\text{B}}_{1}\right]}{K_{s,1}^{{\text{B}}_{1}}} & +\frac{\left[{\text{B}}_{3}\right]}{K_{\text{I},\text{1}}^{{\text{B}}_{3}}}\frac{\left[{\text{B}}_{1}\right]}{K_{s,1}^{{\text{B}}_{1}}} & +\frac{\left[{\text{B}}_{2}\right]}{K_{\text{I},\text{1}}^{{\text{B}}_{2}}}\frac{\left[{\text{B}}_{1}\right]}{K_{s,1}^{{\text{B}}_{1}}} & & \\ & & +\frac{\left[{\text{B}}_{4}\right]}{K_{\text{I},\text{1}}^{{\text{B}}_{4}}}\frac{\left[{\text{B}}_{1}\right]}{K_{s,2}^{{\text{B}}_{1}}} & +\frac{\left[{\text{B}}_{4}\right]}{K_{\text{I},\text{2}}^{{\text{B}}_{4}}}\frac{\left[{\text{B}}_{1}\right]}{K_{s,2}^{{\text{B}}_{1}}} & +\frac{\left[{\text{B}}_{3}\right]}{K_{\text{I},\text{1}}^{{\text{B}}_{3}}}\frac{\left[{\text{B}}_{1}\right]}{K_{s,2}^{{\text{B}}_{1}}} & +\frac{\left[{\text{B}}_{2}\right]}{K_{\text{I},\text{1}}^{{\text{B}}_{2}}}\frac{\left[{\text{B}}_{1}\right]}{K_{s,2}^{{\text{B}}_{1}}} & \\ & & +\frac{\left[{\text{B}}_{4}\right]}{K_{\text{I},\text{1}}^{{\text{B}}_{4}}}\frac{\left[{\text{B}}_{2}\right]}{K_{s,1}^{{\text{B}}_{2}}} & +\frac{\left[{\text{B}}_{4}\right]}{K_{\text{I},\text{2}}^{{\text{B}}_{4}}}\frac{\left[{\text{B}}_{2}\right]}{K_{s,1}^{{\text{B}}_{2}}} & +\frac{\left[{\text{B}}_{3}\right]}{K_{\text{I},\text{1}}^{{\text{B}}_{3}}}\frac{\left[{\text{B}}_{2}\right]}{K_{s,1}^{{\text{B}}_{2}}} & +\frac{\left[{\text{B}}_{2}\right]}{K_{\text{I},\text{1}}^{{\text{B}}_{2}}}\frac{\left[{\text{B}}_{2}\right]}{K_{s,1}^{{\text{B}}_{2}}} & \\ & & & +\frac{\left[{\text{B}}_{4}\right]}{K_{\text{I},\text{1}}^{{\text{B}}_{4}}}\frac{\left[{\text{B}}_{3}\right]}{K_{s,1}^{{\text{B}}_{3}}} & +\frac{\left[{\text{B}}_{4}\right]}{K_{\text{I},\text{2}}^{{\text{B}}_{4}}}\frac{\left[{\text{B}}_{3}\right]}{K_{s,1}^{{\text{B}}_{3}}} & +\frac{\left[{\text{B}}_{3}\right]}{K_{\text{I},\text{1}}^{{\text{B}}_{3}}}\frac{\left[{\text{B}}_{3}\right]}{K_{s,1}^{{\text{B}}_{3}}} & +\frac{\left[{\text{B}}_{2}\right]}{K_{\text{I},\text{1}}^{{\text{B}}_{2}}}\frac{\left[{\text{B}}_{3}\right]}{K_{s,1}^{{\text{B}}_{3}}}, \end{array}$$ (9)

in which $K_{\text{X},i}^{{\text{B}}_{k}}$ denotes the *i*th dissociation constant of the *k*th acceptor, where X is either *s* (dissociation from E⋅B_*i*_) or I (dissociation from an abortive ternary complex).

### Bistability in an open system

It has been observed that bistability can arise when an enzyme is inhibited by one of its substrates in an open system [34], in which substrate enters at a zero-order rate, and exits at a rate that is first-order in the concentration of that substrate. If the substrate can diffuse into the reaction medium according to

$$\begin{array}{c} v_{\text{diff}}=K\left({\left[\text{B}\right]}_{0}-\left[\text{B}\right]\right), \end{array}$$ (10)

where [B]_0_ is the concentration of exogenous substrate, then multiple steady-state solutions for the concentration of substrate can coexist for *v*_enz_ = *v*_diff_. This is illustrated in Fig 3D, where the number of points of intersection of the line (10) with the curve described by Eq (7) will depend on the values of [B]_0_ and the diffusion constant, *K*.

**Fig 4:**
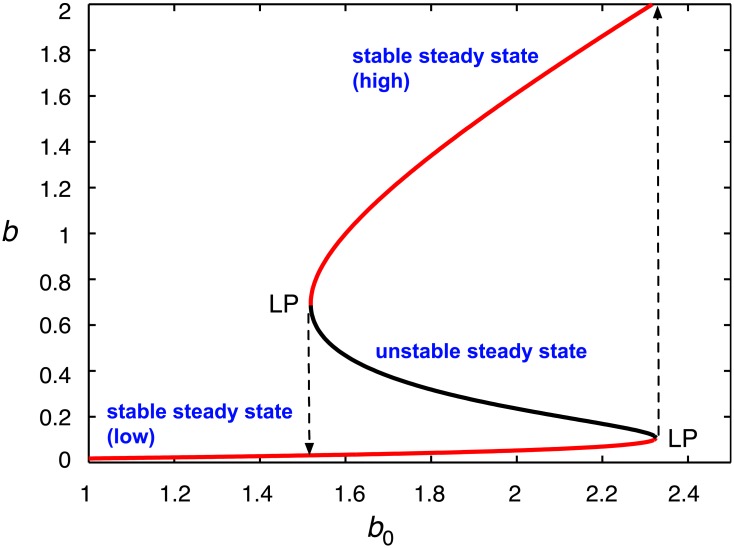
Bistability in an open system for a bisubstrate enzyme reaction exhibiting inhibition at high substrate concentrations. Shown is a bifurcation diagram of the one-dimensional ODE system given by Eq (11), with the external concentration of acceptor substrate, *b*_0_, as the bifurcation parameter. The donor is assumed to be buffered to a constant concentration. The stable steady state levels of the acceptor, *b*, are indicated by the red curves, the black curve denoting the unstable steady state. An exchange of stability between the stable and unstable branches occurs at the limit points (LP), *b*_0_ = 1.518665 and *b*_0_ = 2.325853, as indicated by the dashed lines. Other parameters of the model are given in the text.

Bistability can be demonstrated through numerical simulation of the one-dimensional ODE system:

$$\begin{array}{c} \frac{\text{d}b}{\text{d}t}=K\left(b_{0}-b\right)-\frac{V_{\text{max}}ab}{K_{a}K_{b}+K_{b}a+K_{a}b+\frac{K_{a}}{K_{s}}b^{2}+ab}, \end{array}$$ (11)

where *a* and *b* are the concentrations of the donor and acceptor, respectively. It is assumed that the donor concentration is constant, while the external concentration of *b* is chosen as the parameter to vary. The numerical continuation software AUTO, part of the ODE solver XPPAUT [35], was used to calculate the steady-state level of *b* for increasing *b*_0_. For the parameters *a* = 0.6, *K* = 0.075, *K*_*b*_ = 0.1, *K*_*s*_ = 0.05, *V*_max_ = 1 and *K*_*a*_ = 0.6, bistability is obtained for 1.518665 < *b*_0_ < 2.325853 (Fig 4).

Within this range two stable steady states of acceptor concentration can coexist, as shown by upper and lower branches in *b*–*b*_0_ space. This can be confirmed by solving d*v*_diff_/d*b* = −*K* for *b*, using the parameters of Fig 3D, and computing the ordinate-axis intercept for *v*_diff_ at these two concentrations, which will be points of tangency of the two lines described by Eq (10) with the velocity–substrate curve. The values of *b*, computed in Mathematica (version 11.0.1; Wolfram Research, Inc.), are b* = {0.10755, 0.693546}. Substituting into (10), we evaluate b* + *v*_diff_(b*)/*K* = *b*_0_, obtaining the corresponding solutions *b*_0_ = {1.51866, 2.32585}.

### A model of multiple competing substrates with inhibition at high concentrations

The reaction scheme shown in Fig 2 is modelled with five differential equations,

$$\begin{array}{ccc} \frac{\text{d}b_{1}}{\text{d}t} & = & K\left(b_{0}-b_{1}\right)-\left(v_{1}+v_{2}\right) \end{array}$$ (12)

$$\begin{array}{ccc} \frac{\text{d}b_{2}}{\text{d}t} & = & K\left(b_{0}-b_{2}\right)+v_{1}-v_{3} \end{array}$$ (13)

$$\begin{array}{ccc} \frac{\text{d}b_{3}}{\text{d}t} & = & K\left(b_{0}-b_{3}\right)+v_{2}-v_{4} \end{array}$$ (14)

$$\begin{array}{ccc} \frac{\text{d}b_{4}}{\text{d}t} & = & K\left(b_{0}-b_{4}\right)+v_{3}+v_{4} \end{array}$$ (15)

$$\begin{array}{ccc} \frac{\text{d}a}{\text{d}t} & = & K\left(a_{0}-a\right)-\left(v_{1}+v_{2}+v_{3}+v_{4}\right) \end{array}$$ (16)

where the *b*_*i*_ represent the acceptor concentrations [B_*i*_], *i* = 1 … 3, *a* is the concentration of UDP-Gal, and the enzyme velocities *v*_1_ … *v*_4_ are described by Eq (8). As before, the model assumes free diffusion of substrates into the medium in which enzyme is active [36]. There will be additional terms in $s_{{\text{B}}_{j}}$ and *s*_I_, since there will be two sets of constants for the initial oligosaccharide substrate B_1_, one set for each recognition site. The total enzymic rate of removal of B_1_, for saturating levels of Ax, will be

$$\begin{array}{c} v_{1}+v_{2}=\frac{V_{1}b_{1}}{K_{{\text{m}}_{1}}+b_{1}}+\frac{V_{2}b_{1}}{K_{{\text{m}}_{2}}+b_{1}}. \end{array}$$ (17)

Assuming that the maximal velocities of each of *v*_1_ and *v*_2_ are the same, we can solve for substrate concentration at half-maximal velocity, to obtain apparent *K*_m_ as the geometric mean of the individual Michaelis constants, $K_{\text{m}}^{\text{app}}=\sqrt{K_{{\text{m}}_{1}}K_{{\text{m}}_{2}}}$. Under the same assumption, for a substrate with *m* recognition domains, the apparent *K*_m_ will be the solution to

$$\begin{array}{c} 1=\sum_{i=1}^{m}\frac{K_{\text{m}}^{\text{app}}}{K_{{\text{m}}_{j}}+K_{\text{m}}^{\text{app}}}. \end{array}$$ (18)

**Fig 5:**
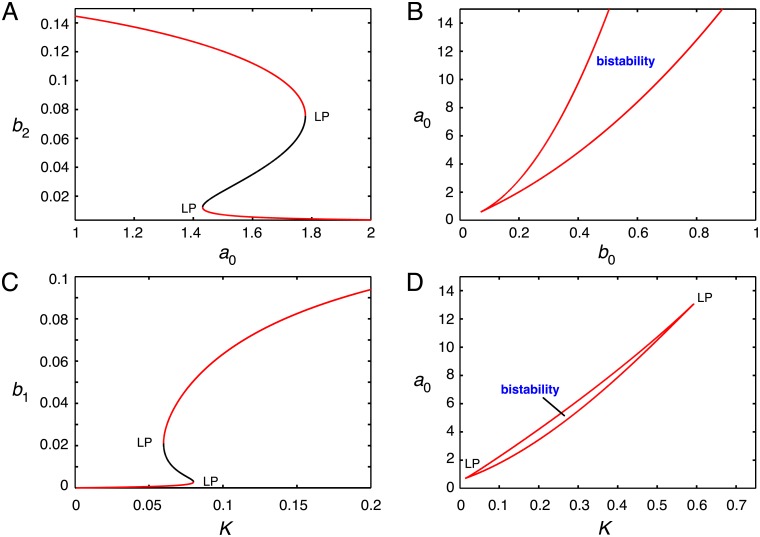
Bistability in an open reaction system described by the ODE system of Eqs (12)–(16), based on the reactions of Fig 2. Parameters of the model are given in Table 1. A. Steady-state levels of *b*_2_ as the external concentration of donor substrate, *a*_0_, is varied, with *b*_0_ = 0.3; limit points (LP) separate the branches of stable (red curve) and unstable (black curve) steady states. B. Cusp in *a*_0_–*b*_0_ space, with branches of limit points enclosing a region of bistability. C. Steady-state levels of initial substrate, *b*_1_, as the diffusion constant, *K*, is varied. D. Region of bistability in *a*_0_–*K* space, with terminal limit points as indicated.

Numerical simulation of the model also displayed bistability (Fig 5). Using a two-parameter continuation, the region of *a*_0_–*b*_0_ space under which bistability exists was determined (Fig 5B). The values of the external concentrations at the point of the cusp were found to be (*b*_0_, *a*_0_) = (0.07094, 0.5959). Bistability was also obtained by varying the diffusion constant, *K* (Fig 5C); a two-parameter continuation in *a*_0_–*K* space revealed a closed region of bistability (Fig 5D).

**Table 1:** Parameters used in the model of multi-substrate competition described by Eqs (12)–(16). Ax and B_*i*_ are, respectively, the donor UDP-Gal, and the oligosaccharide acceptors shown in Fig 2. Maximal velocities of all reactions in the model were set to 5.0 and the value of the diffusion constant (*K*) was 0.075.

	Ax	B_1_	B_2_	B_3_	B_4_
External concentration, [X]_0_	1.5	0.15	0.15	0.15	0.15
Michaelis constants, *K*_m_	n/a	0.25, 0.45	0.45	0.45	n/a
Substrate-inhibition constants, *K*_*I*_	n/a	n/a	0.02	20	20,20
Dissociation constants, *K*_*s*_	0.5	0.05,0.05	0.002	0.1	n/a

## Discussion

In this article a general equation for multi-substrate inhibition is derived, from which are deduced a number of properties of a system of reactions involving rate laws of this kind. Enzymes following a quasi-steady-state compulsory mechanism described by Eq (5) will not show this response with donor concentrations at saturating levels. While the nature of such competitive inhibition had been examined by Schnell and Mendoza [18], and our initial result was presented, without proof, by Umaña and Bailey [12], to our knowledge, this work is the first to present a derivation of a bisubstrate reaction equation with multi-substrate competition, with an extension to include substrate inhibition.

We have extended the treatment of multi-substrate enzymes obeying rapid-equilibrium random-order kinetics to systems exhibiting inhibition at high substrate concentrations. Special note was made of the additional complication of oligosaccharide acceptors, which will have multiple dissociation constants when multivalency is present, and it was shown that, in the bivalent case, an overall Michaelis constant can be predicted from the geometric mean of the individual *K*_m_ values. The work of Degn [34] was applied to the transferase enzymes acting in a system held thermodynamically far from equilibrium, and it was shown that two stable solutions can exist over a range of external substrate concentrations.

Bistability was also shown to be possible for a system of reactions catalysed by the enzyme *β*-*N*-acetylglucosaminylglycopeptide *β*-1,4-galactosyltransferase (GalT) evincing both multi-substrate competition and multivalency. Our model provides a possible explanation for both the compulsory-order catalytic mechanism of this enzyme, reported by Qasba *et al.* [24], in which the donor binds before the acceptor, and the inhibition observed by Freilich *et al.* at high acceptor concentrations [26]. A derivation of the random-order two-substrate mechanism, under quasi-steady-state assumptions, will lead to a 2:2 rational function that is second order with respect to the concentration of acceptor in both the numerator and denominator [37]. Although this non-Michaelian function would give rise to a velocity-substrate curve similar to that observed with substrate inhibition, it conflicts with the available evidence for the catalytic mechanism. If, as we propose here, the acceptor binds as a substrate analog of the donor, at the donor site, followed by a further acceptor-binding step to form a dead-end ternary complex, the apparent paradox is resolved.

As a biological phenomenon, bistability has previously been identified in apoptosis [38], cancer [39], disease progression [40], cell cycling [41], cell motility [42] and differentiation [43]. It has also been reported in an open reconstituted enzyme system containing phosphofructokinase [44]. Multistability is well known in the context of ultrasensitivity [45], and similar phenomena, such as cooperativity and allostery, where enzymes possess switch-like behaviour [46]. In enzyme-kinetic models, a general condition for multistability is that the rate law be a non-monotonic function of the reactant concentrations. Hence, the competitive inhibition introduced by multi-substrate competition is not a necessary, or sufficient, condition for switching behaviour; rather, it is the formation of ternary enzyme-substrate complexes that can lead to non-monotonicity in the enzyme rate law. The first derivative of such a function, possessing at least one maximum or minimum, must undergo a change of sign, as the substrate or effector concentration is varied. Since the property is shown to be possible for a single enzyme, its origin can be distinguished from that based on network topology [47] or feedback regulation [48].

Our main result is consistent with the prediction by Neelamegham and Liu [49] that bistability could arise under circumstances where Michaelis-Menten kinetics, with nonlinearities caused by large numbers of possible substrates and products, were combined with feedback/feedforward regulation. We have considered only initial rate kinetics in this study, ignoring the effects of product concentration in the derivations in the S1 Appendix, although product inhibition effects will also play a role, as can be seen at the early stages of the proof. In neglecting the product concentrations, we have constructed the system in such a way that the primary cause of the bistability is more readily apparent. The models of Shen and Larter [50], who studied the membrane-bound enzyme acetylcholinesterase, not only displayed bistability, but also oscillatory behaviour when either autocatalysis or product inhibition were included. Higher order dynamic behaviour might therefore arise if our model was expanded to incorporate the effects of product concentrations.

The conventional approach to modelling metabolism has involved the construction of systems of ordinary differential equations using kinetic rate laws appropriate to the enzymes and transporters involved, as has been the case for most models of glycosylation to date [12, 14, 51], and in the present work. Such models assume an underlying deterministic process and a detailed knowledge of the parameters, which may not be available. Another approach is to model the transitions between the reactants in a network by a Markov chain, an application of which to glycosylation has recently appeared [52]. It is known that bistability can arise within noise-driven biochemical systems operating at the level of cellular volume [53], even where it is not predicted in the deterministic limit. Multistability as a general principle, therefore, and outside of the specific application to glycosylation, can exist within either modelling framework. As the volume size of the system decreases, the rate of switching between biologically realisable steady states increases exponentially [54], which has implications for several of the phenomena cited above, such as cellular differentiation and cancer. The transitions between steady states, perturbed by stochastic fluctuations, may additionally require that the system be close to the boundaries of the basins of attraction [55].

### Conclusion

These results demonstrate that the complex interplay of enzyme and substrate can give rise to nonlinear behaviour in systems of reactions held far from thermodynamic equilibrium. The significance of the present study is that small changes in one condition, such as the amount of available sugar-nucleotide donor [56], might incur large and abrupt changes in the amount of product formed. Since GalT action influences the number of sites available for sialylation, such changes should have important implications for cancer progression and metastasis, which have been shown to be related to these processes [57], and for biotechnology, such as in the production of therapeutic antibodies [58], which can be influenced through control of metabolic flux [59]. More generally, the occurrence of bistability in metabolism could provide the basis for cellular long-term memory [60]. The commonly occurring pattern of substrate inhibition in transferases should complement the already known behaviours of models based on sigmoidal functions. For instance, it is known that different glycosylation enzymes associate, and co-locate with the Golgi, according to the ‘kin recognition’ model [61], and may therefore display cooperativity. Whether a combination of cooperativity and substrate inhibition could lead to higher order dynamic behaviour, such as oscillations in acceptor concentration, is an open question that deserves further study.

## Supporting information

S1 Appendix (PDF)
