# Supplementary material for: A mechanism for bistability in glycosylation
Source: PLoS Comput Biol. 2018 Aug 3;14(8):e1006348. doi: 10.1371/journal.pcbi.1006348 (PMC6093706; doi:10.1371/journal.pcbi.1006348)
Supplement: S1 Appendix — (PDF) [file pcbi.1006348.s001.pdf]

S1 Appendix. Ancillary derivations.

Derivation of a rapid equilibrium bisubstrate kinetic rate law with multiple-substrate competition under rapid-equilibrium assumptions

For the mechanism shown in Fig 1A, and assuming rapid release of product(s), the initial rate will depend linearly on the concentration of enzyme-substrate complex,

$$v_j = k_j[\mathrm{E{\cdot}Ax{\cdot}B}_j].$$

The total enzyme concentration, when n acceptor-substrates are in competition, is

$$[\mathrm{E_0}] = [\mathrm{E}] + [\mathrm{E{\cdot}Ax}] + \sum_{i=1}^{n}[\mathrm{E{\cdot}B}_i] + \sum_{i=1}^{n}[\mathrm{E{\cdot}Ax{\cdot}B}_i] + \sum_{i=1}^{n}[\mathrm{E{\cdot}A{\cdot}Bx}_i] + \sum_{i=1}^{n}[\mathrm{E{\cdot}Bx}_i] + [\mathrm{E{\cdot}A}].$$

The dissociation constants given in Fig 1A have the following definitions:

$$\begin{aligned}
K_s^{\mathrm{Ax}} &= \frac{[\mathrm{E}][\mathrm{Ax}]}{[\mathrm{E{\cdot}Ax}]} & K_s^{\mathrm{B}_j} &= \frac{[\mathrm{E}][\mathrm{B}_j]}{[\mathrm{E{\cdot}B}_j]}\\
K_{\mathrm{m}}^{\mathrm{B}_j} &= \frac{[\mathrm{E{\cdot}Ax}][\mathrm{B}_j]}{[\mathrm{E{\cdot}Ax{\cdot}B}_j]} & K_{\mathrm{m}_j}^{\mathrm{Ax}} &= \frac{[\mathrm{E{\cdot}B}_j][\mathrm{Ax}]}{[\mathrm{E{\cdot}Ax{\cdot}B}_j]}\\
K_s^{\mathrm{A}} &= \frac{[\mathrm{E}][\mathrm{A}]}{[\mathrm{E{\cdot}A}]} & K_s^{\mathrm{Bx}_j} &= \frac{[\mathrm{E}][\mathrm{Bx}_j]}{[\mathrm{E{\cdot}Bx}_j]}\\
K_{\mathrm{m}}^{\mathrm{Bx}_j} &= \frac{[\mathrm{E{\cdot}A}][\mathrm{Bx}_j]}{[\mathrm{E{\cdot}A{\cdot}Bx}_j]} & K_{\mathrm{m}_j}^{\mathrm{A}} &= \frac{[\mathrm{E{\cdot}Bx}_j][\mathrm{A}]}{[\mathrm{E{\cdot}A{\cdot}Bx}_j]}
\end{aligned}$$

We aim to express the total enzyme concentration in terms of the concentration of a common enzyme-substrate complex, E·Ax. For example, using the definitions of K_s^{Ax} and $K_s^{\mathrm{B}_j}$, we see that

$$[\mathrm{E{\cdot}B}_i] = [\mathrm{E{\cdot}Ax}]\frac{K_s^{\mathrm{Ax}}}{[\mathrm{Ax}]}\frac{[\mathrm{B}_i]}{K_s^{\mathrm{B}_i}}$$

and therefore,

$$[\mathrm{E_0}] = [\mathrm{E{\cdot}Ax}]\left(\frac{K_s^{\mathrm{Ax}}}{[\mathrm{Ax}]} + 1 + \frac{K_s^{\mathrm{Ax}}}{[\mathrm{Ax}]}\sum_{i=1}^{n}\frac{[\mathrm{B}_i]}{K_s^{\mathrm{B}_i}} + \sum_{i=1}^{n}\frac{[\mathrm{B}_i]}{K_{\mathrm{m}}^{\mathrm{B}_i}} + \frac{K_s^{\mathrm{Ax}}}{[\mathrm{Ax}]}\frac{[\mathrm{A}]}{K_s^{\mathrm{A}}}\sum_{i=1}^{n}\frac{[\mathrm{Bx}_i]}{K_{\mathrm{m}}^{\mathrm{Bx}_i}} + \frac{K_s^{\mathrm{Ax}}}{[\mathrm{Ax}]}\sum_{i=1}^{n}\frac{[\mathrm{Bx}_i]}{K_s^{\mathrm{Bx}_i}} + \frac{[\mathrm{A}]}{K_s^{\mathrm{A}}}\frac{K_s^{\mathrm{Ax}}}{[\mathrm{Ax}]}\right).$$

The rate of appearance of the jth product will then be

$$v_j = \frac{k_j[\mathrm{E_0}][\mathrm{B}_j]/K_{\mathrm{m}}^{\mathrm{B}_j}}{\frac{K_s^{\mathrm{Ax}}}{[\mathrm{Ax}]} + 1 + \frac{K_s^{\mathrm{Ax}}}{[\mathrm{Ax}]}\sum_{i=1}^{n}\frac{[\mathrm{B}_i]}{K_s^{\mathrm{B}_i}} + \sum_{i=1}^{n}\frac{[\mathrm{B}_i]}{K_{\mathrm{m}}^{\mathrm{B}_i}} + \frac{[\mathrm{A}]}{K_s^{\mathrm{A}}}\frac{K_s^{\mathrm{Ax}}}{[\mathrm{Ax}]}\sum_{i=1}^{n}\frac{[\mathrm{Bx}_i]}{K_{\mathrm{m}}^{\mathrm{Bx}_i}} + \frac{K_s^{\mathrm{Ax}}}{[\mathrm{Ax}]}\sum_{i=1}^{n}\frac{[\mathrm{Bx}_i]}{K_s^{\mathrm{Bx}_i}} + \frac{[\mathrm{A}]}{K_s^{\mathrm{A}}}\frac{K_s^{\mathrm{Ax}}}{[\mathrm{Ax}]}}$$

Assuming initial-rate kinetics, we set the product concentrations to zero:

$$v_j = \frac{k_j[\mathrm{E}_0][\mathrm{B}_j]/K_\mathrm{m}^{\mathrm{B}_j}}{\dfrac{K_s^\mathrm{Ax}}{[\mathrm{Ax}]} + 1 + \dfrac{K_s^\mathrm{Ax}}{[\mathrm{Ax}]}\displaystyle\sum_{i=1}^{n}\frac{[\mathrm{B}_i]}{K_s^{\mathrm{B}_i}} + \sum_{i=1}^{n}\frac{[\mathrm{B}_i]}{K_\mathrm{m}^{\mathrm{B}_i}}}.$$

Defining the maximum velocity as $V_j = k_j[\mathrm{E}_0]$, and multiplying both numerator and denominator by $K_\mathrm{m}^{\mathrm{B}_j}[\mathrm{Ax}]$, we obtain

$$v_j = \frac{V_j[\mathrm{Ax}][\mathrm{B}_j]}{K_s^\mathrm{Ax}K_\mathrm{m}^{\mathrm{B}_j}\left(1 + \displaystyle\sum_{i=1}^{n}\frac{[\mathrm{B}_i]}{K_s^{\mathrm{B}_i}}\right) + K_\mathrm{m}^{\mathrm{B}_j}[\mathrm{Ax}]\left(1 + \displaystyle\sum_{i=1}^{n}\frac{[\mathrm{B}_i]}{K_\mathrm{m}^{\mathrm{B}_i}}\right)}. \tag{S1}$$

Defining $s_{\mathrm{B}_j} = \sum_{i\neq j}\frac{[\mathrm{B}_i]}{K_\mathrm{m}^{\mathrm{B}_i}}$, $s'_{\mathrm{B}_j} = \sum_{i\neq j}\frac{[\mathrm{B}_i]}{K_s^{\mathrm{B}_i}}$, and rearranging,

$$v_j = \frac{V_j[\mathrm{Ax}][\mathrm{B}_j]}{K_s^\mathrm{Ax}K_\mathrm{m}^{\mathrm{B}_j}\left(1 + s'_{\mathrm{B}_j}\right) + K_\mathrm{m}^{\mathrm{B}_j}[\mathrm{Ax}](1 + s_{\mathrm{B}_j}) + K_s^\mathrm{Ax}\dfrac{K_\mathrm{m}^{\mathrm{B}_j}}{K_s^{\mathrm{B}_j}}[\mathrm{B}_j] + [\mathrm{Ax}][\mathrm{B}_j]}. \tag{S2}$$

From the observation that the product of the equilibrium constants of any sequence of equilibria that form a closed cycle is equal to one (also known as the Wegscheider condition [1]), we see that $K_{\mathrm{m}_j}^\mathrm{Ax} = K_s^\mathrm{Ax}K_\mathrm{m}^{\mathrm{B}_j}/K_s^{\mathrm{B}_j}$.

Derivation of a compulsory-order bisubstrate kinetic rate law with multiple-substrate competition under quasi-steady-state assumptions

For the mechanism shown in Fig 1B, the initial rate of appearance of product(s), will be

$$v_j = k_{3_j}[\mathrm{E{\cdot}Ax{\cdot}B}_j].$$

Following the method of Volkenstein and Goldstein [2], we treat the scheme as a directed graph (digraph). For each enzyme species, we collect all of the acyclic paths in the figure leading to that species, in which each node in the graph has been traversed only once. For the species E, three "basic trees" can be constructed, with two for E·Ax and one for E·Ax·B_j. The sum of the products of the rate constants and associated concentrations of Ax and B_j, in each basic tree, form what is called the "determinant" of that enzyme species.

E:

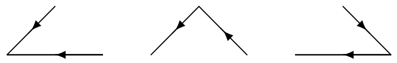

$$D_1 = k_{-1}\sum_{i=1}^{n}(k_{3_i} + k_{-2_i}) + \sum_{i=1}^{n} k_{2_i}k_{3_i}[\mathrm{B}_i]$$

E·Ax:

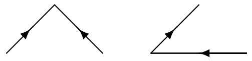

$$D_2 = k_1[\mathrm{Ax}]\sum_{i=1}^{n}(k_{3_i} + k_{-2_i})$$

E·Ax·B_j:

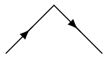

$$D_3 = k_1k_{2_j}[\mathrm{Ax}][\mathrm{B}_j]$$

The rate of product formation at steady state will be given by Mason's gain formula [3]:

$$\begin{aligned} v_j &= [\mathrm{E}_0]\frac{k_{3_j}D_3}{D_1 + D_2 + D_3} \\ &= [\mathrm{E}_0]\frac{k_{3_j}(k_1k_{2_j}[\mathrm{Ax}][\mathrm{B}_j])}{k_{-1}\sum_{i=1}^{n}(k_{3_i} + k_{-2_i}) + \sum_{i=1}^{n} k_{2_i}k_{3_i}[\mathrm{B}_i] + k_1[\mathrm{Ax}]\sum_{i=1}^{n}(k_{3_i} + k_{-2_i}) + k_1k_{2_j}[\mathrm{Ax}][\mathrm{B}_j]}. \end{aligned}$$

Dividing by $k_1k_{2_j}$ and gathering like terms, we have

$$v_j = \frac{V_j[\mathrm{Ax}][\mathrm{B}_j]}{K_s^{\mathrm{Ax}}K_{\mathrm{m}}^{\mathrm{B}_j}(1 + s_{\mathrm{B}_j}) + K_{\mathrm{m}}^{\mathrm{B}_j}[\mathrm{Ax}] + K_{\mathrm{m}_j}^{\mathrm{Ax}}[\mathrm{B}_j] + [\mathrm{Ax}][\mathrm{B}_j]} \tag{S3}$$

where $V_j = k_{3_j}[\mathrm{E}_0]$, $K_s^{\mathrm{Ax}} = \dfrac{k_{-1}}{k_1}$, $K_{\mathrm{m}}^{\mathrm{B}_j} = \dfrac{\sum_{i=1}^{n}(k_{3_i} + k_{-2_i})}{k_{2_j}}$, $K_{\mathrm{m}_j}^{\mathrm{Ax}} = \dfrac{k_{3_j}}{k_1}$ and $s_{\mathrm{B}_j} = \dfrac{\sum_{i \neq j} k_{2_i}k_{3_i}[\mathrm{B}_i]}{\sum_{i=1}^{n}(k_{3_i} + k_{-2_i})}$.

References

1. Schuster S, Schuster R. A generalization of Wegscheider's condition. Implications for properties of steady states and for quasi-steady-state approximation. J Math Chem. 1989;3:25–42.

2. Volkenstein MV, Goldstein BN. A new method for solving the problems of the stationary kinetics of enzymological reactions. Biochim Biophys Acta. 1966;115:471–477.

3. Mason SJ. Feedback theory—further properties of signal flow graphs. Proc IRE. 1956;44:920–926.
